# Androgen Imbalance, Cardiovascular-Kidney-Metabolic Syndrome Stages, and Mortality in Men

**DOI:** 10.1016/j.jacadv.2026.103431

**Published:** 2026-11-05

**Authors:** Kaiyuan Liu, Yinman Wang, Zhixiang Wang, Shuning Zhang, Kang Yao, Junbo Ge

**Affiliations:** a Department of Cardiology, Zhongshan Hospital, Fudan University, Shanghai Institute of Cardiovascular Diseases, Shanghai, China; b State Key Laboratory of Cardiovascular Diseases, Zhongshan Hospital, Fudan University, Shanghai, China; c National Clinical Research Center for Interventional Medicine, Shanghai, China

**Keywords:** adiposity, calculated free testosterone, epidemiology, NHANES, sex hormone-binding globulin, total testosterone

## Abstract

**Background:**

Androgen measures reflect adiposity, metabolic health, and systemic illness, but their associations with cardiovascular-kidney-metabolic (CKM) syndrome and mortality may differ across measures and combined profiles.

**Objectives:**

The objective of the study was to examine associations of total testosterone (TT), sex hormone-binding globulin (SHBG), calculated free testosterone (cFT), and joint TT-SHBG phenotypes with prevalent advanced CKM syndrome and mortality.

**Methods:**

We analyzed men aged ≥20 years in the National Health and Nutrition Examination Survey 2013 to 2016 using survey-weighted logistic (odds ratio [OR]) and Cox (hazard ratio [HR]) regression models. Advanced CKM syndrome comprised determinate National Health and Nutrition Examination Survey–adapted stages 3 to 4; cFT was calculated using the Vermeulen equation. Analyses assessed adiposity adjustment and mortality robustness.

**Results:**

Among 5,505 men, 4,005 had determinate CKM status. Low measured TT was associated with prevalent advanced CKM syndrome (OR: 1.78; 95% CI: 1.31-2.42), but the association was attenuated after adjustment for body mass index (OR: 1.27; 95% CI: 0.90-1.79) or waist circumference (OR: 1.22; 95% CI: 0.85-1.75), suggesting that adiposity may partly account for this association. Low TT and cFT measures showed no consistent association with all-cause mortality. In exploratory analyses, the low-TT/high-SHBG phenotype was associated with higher all-cause mortality (HR: 2.06; 95% CI: 1.18-3.58).

**Conclusions:**

Low measured TT was associated with prevalent advanced CKM syndrome, but this association was strongly adiposity-dependent. The low-TT/high-SHBG profile showed an exploratory mortality association not observed for TT or cFT alone and may represent a distinct systemic-vulnerability phenotype. These findings underscore the noninterchangeability of androgen measures and support prospective evaluation of combined TT-SHBG profiles.

Cardiovascular-kidney-metabolic (CKM) syndrome provides an integrated framework for the interrelated burden of adiposity, metabolic dysfunction, chronic kidney disease (CKD), and cardiovascular disease (CVD).^1^, ^2^ Rather than treating these conditions as isolated entities, the CKM framework emphasizes their shared pathophysiology across a continuum from early metabolic risk to established CVD. CKM stages are highly prevalent among U.S. adults,^3^, ^4^, ^5^, ^6^, ^7^, ^8^ and identifying accessible circulating measures associated with heterogeneity across this continuum may help characterize disease burden and prognosis.

In men, androgen status is closely related to cardiometabolic health. Testosterone contributes to body composition, skeletal muscle maintenance, visceral adiposity, insulin sensitivity, vascular function, inflammatory signaling, and physical performance.^9^, ^10^ Lower total testosterone (TT) has been associated with obesity, metabolic syndrome, insulin resistance, diabetes, and adverse cardiovascular profiles.^11^, ^12^, ^13^, ^14^ However, circulating TT and sex hormone-binding globulin (SHBG) are also influenced by age, adiposity, hepatic and nutritional status, and chronic illness, making their interpretation within CKM potentially complex.^15^, ^16^, ^17^

Measurement choice may therefore be important. TT, SHBG, and calculated free testosterone (cFT) represent related but noninterchangeable aspects of androgen physiology.^18^, ^19^, ^20^, ^21^ Although the TT-to-SHBG ratio does not reliably estimate free testosterone in adult men, joint consideration of TT and SHBG may capture a hormonal and systemic-health profile distinct from cFT.^18^, ^19^, ^20^, ^21^ In particular, a low-TT/high-SHBG profile may reflect the convergence of low circulating testosterone with SHBG-related signals of aging, nutritional status, chronic illness, and broader health status, thereby identifying a phenotype of greater systemic vulnerability rather than simply serving as a surrogate for reduced free testosterone.^15^, ^17^, ^22^, ^23^, ^24^, ^25^, ^26^

Few studies have compared multiple androgen measures within the CKM staging framework while accounting for adiposity and evaluating linked mortality. We therefore examined associations of a single measured TT concentration, SHBG, albumin-based cFT, and joint TT-SHBG phenotypes with prevalent National Health and Nutrition Examination Survey (NHANES)–adapted CKM stages and mortality among U.S. adult men. We hypothesized that different androgen measures and combined profiles would show heterogeneous associations with CKM burden and mortality.

## Methods

### Study design and population

We used data from the 2013 to 2014 and 2015 to 2016 cycles of the NHANES, a nationally representative cross-sectional survey of the noninstitutionalized U.S. civilian population. These cycles were selected because TT and SHBG were measured in both. Men aged 20 years or older were eligible.

The combined two-cycle complex survey design was specified before restrictions based on age, sex, hormone availability, CKM status, mortality eligibility, or complete covariate data. Analytic populations were subsequently defined as survey domains to preserve the original strata and primary sampling units. CKM syndrome and mortality analyses used outcome-specific eligible samples, as shown in Figure 1.

**Figure 1:**
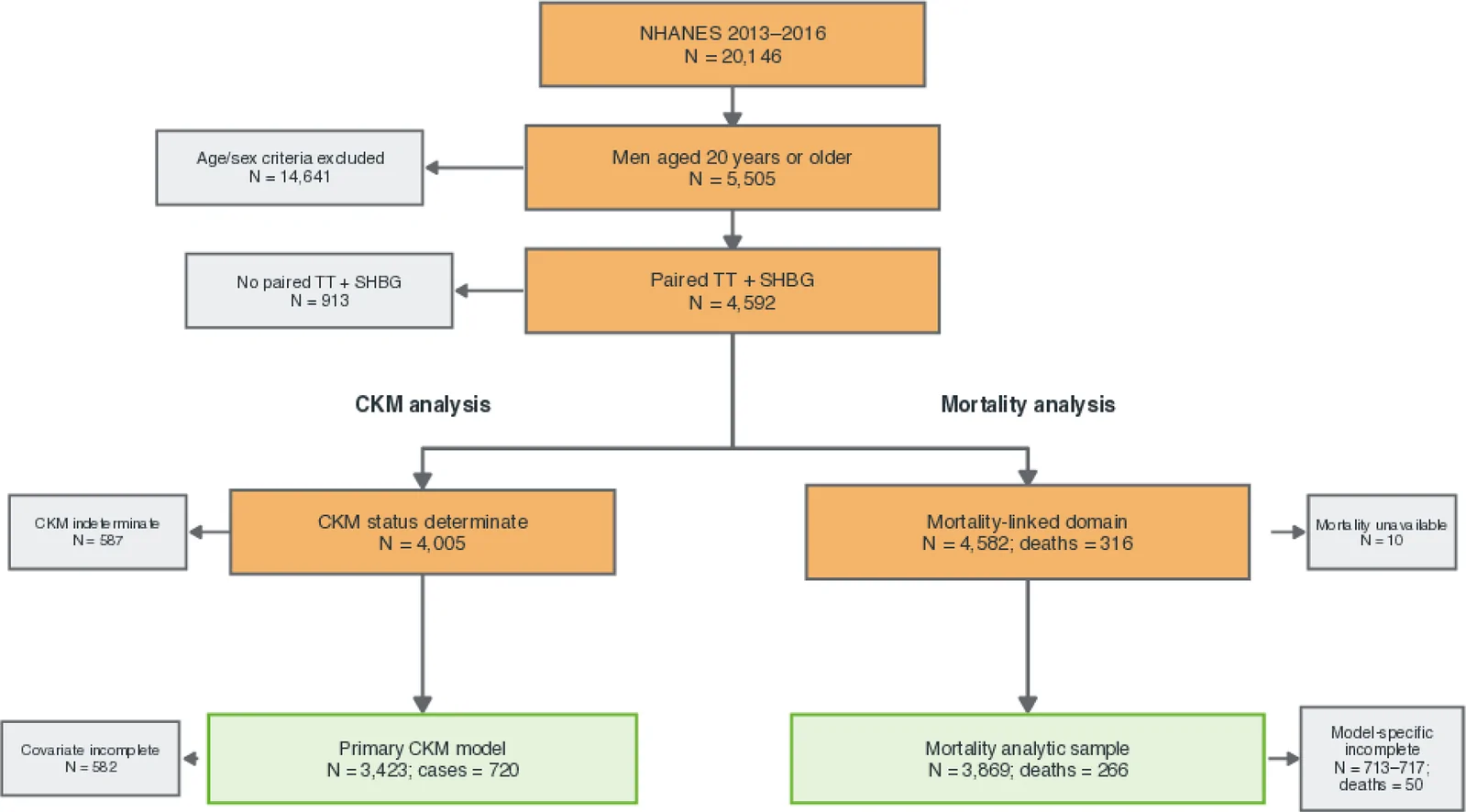
**Participant Selection and Analytic Samples** Unweighted participant counts are shown for NHANES 2013 to 2016. Of the 20,146 participants, 5,505 were men aged 20 years or older; TT was available for 5,000, paired TT and SHBG for 4,592, and calculated free testosterone for 4,588. CKM stage was determinate for 4,005 men in the paired-hormone domain, and the mortality-linked domain included 4,582 men and 316 deaths. The main advanced-CKM model included 3,423 men and 720 cases; available-sample mortality models included 3,865-3,869 men and 266 deaths. CKM = cardiovascular-kidney-metabolic syndrome; NHANES = National Health and Nutrition Examination Survey; SHBG = sex hormone-binding globulin; TT = total testosterone.

### Ethics

The NHANES protocols for the 2013 to 2016 cycles were approved by the National Center for Health Statistics Research Ethics Review Board (Protocol No. 2011-17), and all participants provided written informed consent. This secondary analysis used publicly available, deidentified data and required no additional institutional review board approval.

### Androgen measures

Serum TT and SHBG were measured according to standardized NHANES laboratory protocols.^27^ TT was analyzed in ng/dL and converted to nmol/L when required. A TT concentration below 300 ng/dL was classified as a single low measured concentration for epidemiologic analysis rather than a clinical diagnosis of hypogonadism.^28^, ^29^, ^30^ SHBG was examined continuously, flexibly, and using an exploratory threshold of >57 nmol/L.^27^

The TT-to-SHBG ratio was calculated as 100 × TT (nmol/L)/SHBG (nmol/L). A low ratio was defined as the survey-weighted first quartile in the paired-hormone domain (26.41 ratio units). Because this ratio does not reliably estimate free testosterone in adult men, it was interpreted as a composite TT-SHBG measure.^18^, ^19^, ^20^, ^21^

cFT was derived from TT, SHBG, and measured albumin using the Vermeulen mass-action equation.^20^ cFT was modeled continuously per 0.1 nmol/L and categorically using its survey-weighted first quartile (0.186 nmol/L). A threshold below 0.220 nmol/L and a fixed-albumin calculation were evaluated in sensitivity analyses.^31^

Four mutually exclusive TT-SHBG phenotypes were defined: normal TT/nonhigh SHBG, low TT/nonhigh SHBG, normal TT/high SHBG, and low TT/high SHBG. The normal TT/nonhigh SHBG group served as the reference. Joint-phenotype analyses were considered exploratory.

### NHANES-adapted CKM staging

CKM stage was reconstructed from NHANES questionnaire, examination, and laboratory data according to the American Heart Association framework using tristate component logic.^1^, ^2^ Missing components were not assumed to be absent.

Stage 4 required reported clinical CVD. Among participants without clinical CVD, stage 3 required either very-high-risk CKD or a PREVENT-estimated 10-year total CVD risk of at least 20%. Stage 2 included hypertension, diabetes, CKD, nonfasting triglycerides of at least 135 mg/dL, or operational metabolic syndrome in the absence of a higher stage. Stage 1 included overweight or obesity, abdominal obesity, or prediabetes in the absence of stages 2 to 4. Stage 0 required the documented absence of all higher-stage and stage-1 components; otherwise, CKM stage was classified as indeterminate.

The PREVENT base 10-year total CVD equation was implemented using published sex-specific coefficients and applicability criteria.^32^ Risk was estimated only for participants aged 30 to 79 years without clinical CVD who had all required inputs within the published ranges. Input values were not truncated or capped, and adults aged 80 years or older were not assigned an age of 79 years in the primary definition.

Because NHANES lacked the imaging and complete biomarker data required to identify the full spectrum of subclinical CVD, the reconstructed stages are described as NHANES-adapted CKM stages. The primary cross-sectional outcome was prevalent advanced CKM, defined as determinate stages 3 to 4 vs determinate stages 0 to 2. Stage 3, stage 4, very-high-risk CKD, PREVENT risk of at least 20%, and clinical CVD were examined separately as exploratory outcomes.

### Mortality outcomes

Mortality status was obtained from the 2019 public-use National Center for Health Statistics Linked Mortality File, with follow-up through December 31, 2019. Follow-up extended from the mobile examination center examination date until death or administrative censoring.

All-cause mortality was the principal mortality outcome. Cardiovascular mortality was exploratory and included public-use leading-cause categories for heart disease and cerebrovascular disease. Deaths with unavailable cause were retained in the all-cause mortality analysis but were not classified as noncardiovascular deaths.

### Covariates and analytic models

The primary adjustment set included age, race and ethnicity, education, poverty-income ratio, smoking status, alcohol use, moderate-to-vigorous physical activity, sedentary time, and NHANES cycle. Invalid and special response codes were treated as missing. Age was modeled using a restricted cubic spline with knots at the survey-weighted 5th, 35th, 65th, and 95th percentiles.

Body mass index (BMI) and waist circumference were added separately to assess the influence of overall and central adiposity on CKM associations. Shared-complete-case analyses were used to distinguish attenuation related to covariate adjustment from changes caused by sample restriction. Given the cross-sectional design, these analyses were not interpreted as formal mediation analyses.

Mortality associations were first estimated in the largest available sample without CKM stage adjustment. Secondary analyses were restricted to participants with determinate CKM stage and were subsequently adjusted for CKM stage, allowing the effects of sample restriction and CKM conditioning to be evaluated separately.

### Statistical analysis

All analyses incorporated NHANES survey weights, strata, and primary sampling units. Primary analyses used the mobile examination center weight divided by 2 for the 2 combined survey cycles. The full survey design was specified before domain subsetting.

Continuous variables were summarized using survey-weighted mean ± SE or median (25th-75th percentile [Q1-Q3]), as appropriate. Categorical variables were summarized using unweighted counts and survey-weighted percentages. Design-based Wald or Rao-Scott F-adjusted tests were used for comparisons across CKM stages.

Survey-weighted quasibinomial logistic regression estimated ORs and 95% CIs for prevalent CKM outcomes. Survey-weighted Cox proportional hazards regression estimated HRs and 95% CIs for mortality outcomes. TT and SHBG were entered jointly in continuous models. Restricted cubic splines were used to assess potential nonlinear associations.

Sensitivity analyses addressed adiposity, albumin specification, chronic illness, functional limitation, morning examination session, hormone-relevant medications, early deaths, tail-restricted flexible analyses, and alternative CKM and cFT specifications. Proportional-hazards diagnostics, influential observations, missing-data assessment, survey-design implementation, and additional spline details are described in the Supplemental Methods.

The association between low measured TT and prevalent advanced CKM was designated as primary. cFT associations with advanced CKM and TT or cFT associations with all-cause mortality were secondary. Analyses involving SHBG categories, the TT-to-SHBG ratio, joint TT-SHBG phenotypes, stage-specific outcomes, cardiovascular mortality, spline models, and sensitivity analyses were exploratory. Benjamini-Hochberg false discovery rate (FDR) correction (q-value <0.05) was applied within defined exploratory analysis families.

Analyses were performed in R (version 4.4.2; R Foundation for Statistical Computing) using the survey package (version 4.4.2) and survival package (version 3.7.0).

## Results

### Study population and characteristics

Among 20,146 participants in NHANES 2013 to 2016, 11,488 were adults aged 20 years or older and 5,505 were men. TT was available for 5,000 men, paired TT and SHBG measurements for 4,592, and cFT for 4,588. CKM stage was determinate for 4,005 men in the paired-hormone domain. After exclusion of 10 participants without mortality-linkage eligibility or known mortality status, 4,582 men were included in the mortality-linked domain (Figure 1).

Table 1 included 3,974 men with complete descriptive variables. The survey-weighted distribution was 24.6% for CKM stages 0 to 1, 59.4% for stage 2, 5.3% for stage 3, and 10.7% for stage 4. Participants at higher CKM stages were generally older and had greater adiposity and cardiometabolic burden, accompanied by differences in TT, SHBG, and cFT. Supplementary descriptive analyses showed that the androgen measures were correlated but identified partly overlapping participant groups (Supplemental Tables 10 and 11).

**Table 1:** Participant Characteristics by CKM Stage

	Overall (N = 3,974, 100.0%)	Stage 0-1 (n = 852, 24.6%)	Stage 2 (n = 2,275, 59.4%)	Advanced CKM (Stage 3-4) (n = 847, 16.0%)	*P* Value
Age, y	46.3 (0.4)	37.3 (0.8)	44.7 (0.4)	66.3 (0.5)	<0.001
Race and ethnicity, n (weighted %)					**<0.001**
Mexican American	623 (9.5)	112 (9.0)	398 (10.8)	113 (5.7)	
Other Hispanic	434 (6.3)	89 (6.2)	279 (7.1)	66 (3.3)	
Non-Hispanic White	1,516 (65.6)	334 (66.4)	795 (63.5)	387 (72.1)	
Non-Hispanic Black	758 (9.6)	171 (10.9)	396 (8.8)	191 (10.8)	
Non-Hispanic Asian	503 (5.6)	119 (5.4)	320 (6.0)	64 (4.0)	
Other/multiracial	140 (3.5)	27 (2.1)	87 (3.8)	26 (4.2)	
Education, n (weighted %)					**0.004**
<9th grade	396 (5.5)	52 (4.7)	221 (5.2)	123 (7.4)	
9th-11th grade	524 (9.8)	107 (9.0)	300 (9.6)	117 (11.8)	
High school graduate or equivalent	930 (22.7)	197 (22.3)	524 (22.4)	209 (24.6)	
Some college or associate degree	1,098 (29.8)	223 (25.7)	645 (30.9)	230 (31.8)	
College graduate or above	1,025 (32.2)	273 (38.3)	585 (31.8)	167 (24.4)	
Poverty income ratio	3.10 [1.56, 5.00]	3.00 [1.59, 5.00]	3.28 [1.64, 5.00]	2.51 [1.34, 4.13]	<0.001
Smoking status, n (weighted %)					**<0.001**
Never	1903 (50.6)	497 (58.9)	1,128 (51.8)	278 (33.3)	
Former	1,154 (29.0)	169 (23.1)	616 (27.4)	369 (43.8)	
Current	916 (20.5)	186 (18.0)	531 (20.8)	199 (22.9)	
Any alcohol use, n (weighted %)	3,095 (86.0)	648 (84.4)	1792 (87.1)	655 (84.2)	0.20
Moderate-to-vigorous physical activity, min/week	315 [60, 1,080]	405 [125, 1,320]	360 [70, 1,200]	105 [0, 480]	<0.001
Sedentary time, min/day	360 [240, 540]	360 [240, 540]	360 [240, 540]	420 [300, 540]	0.045
Total testosterone, ng/dL	396 [301, 515]	472 [358, 597]	378 [288, 489]	356 [269, 486]	<0.001
Sex hormone-binding globulin, nmol/L	37.2 [27.0, 52.6]	39.2 [29.5, 54.4]	34.4 [24.6, 47.3]	50.5 [35.2, 69.4]	<0.001
TT-to-SHBG ratio, 100 x nmol/L per nmol/L	36.1 [27.1, 49.0]	40.6 [30.1, 53.7]	37.9 [29.0, 50.6]	25.1 [19.2, 31.8]	<0.001
Calculated free testosterone, nmol/L	0.241 [0.189, 0.308]	0.282 [0.220, 0.362]	0.241 [0.196, 0.305]	0.185 [0.146, 0.233]	<0.001
Body mass index, kg/m^2^	28.6 (0.2)	26.1 (0.2)	29.2 (0.2)	30.3 (0.3)	<0.001
Waist circumference, cm	101.2 (0.5)	92.6 (0.5)	102.7 (0.5)	109.3 (0.9)	<0.001
Hypertension, n (weighted %)	2,199 (51.4)	0 (0.0)	1,477 (64.3)	722 (82.8)	<0.001
Diabetes, n (weighted %)	758 (14.8)	0 (0.0)	339 (12.8)	419 (45.0)	<0.001
Chronic kidney disease, n (weighted %)	610 (11.0)	0 (0.0)	204 (7.7)	406 (41.0)	<0.001
Clinical cardiovascular disease, n (weighted %)	557 (10.7)	0 (0.0)	0 (0.0)	557 (67.0)	<0.001

Values are unweighted n (survey-weighted %), survey-weighted mean (SE), or survey-weighted median (Q1, Q3); percentages use variable-specific nonmissing denominators. *P* values are design-based omnibus comparisons (Wald F tests for weighted means or pooled midranks; Rao-Scott F-adjusted Pearson tests for categorical variables).

CKM = cardiovascular-kidney-metabolic syndrome; SHBG = sex hormone-binding globulin; TT = total testosterone.

### Androgen measures and prevalent CKM

The primary analysis included 3,423 men with 720 cases of prevalent advanced CKM. Low measured TT was associated with higher odds of advanced CKM after adjustment for demographic and lifestyle factors (OR: 1.78; 95% CI: 1.31-2.42; *P* = 0.002). The association was attenuated after additional adjustment for BMI (OR: 1.27; 95% CI: 0.90-1.79; *P* = 0.15; N/cases = 3,404/703) or waist circumference (OR: 1.22; 95% CI: 0.85-1.75; *P* = 0.24; N/cases = 3,350/668). In 3,343 shared complete cases, the corresponding ORs were 1.76 (95% CI: 1.25-2.46), 1.22 (95% CI: 0.84-1.78), and 1.22 (95% CI: 0.85-1.75), respectively, indicating that the attenuation was not explained by changes in the analytic sample (Figure 2).

**Figure 2:**
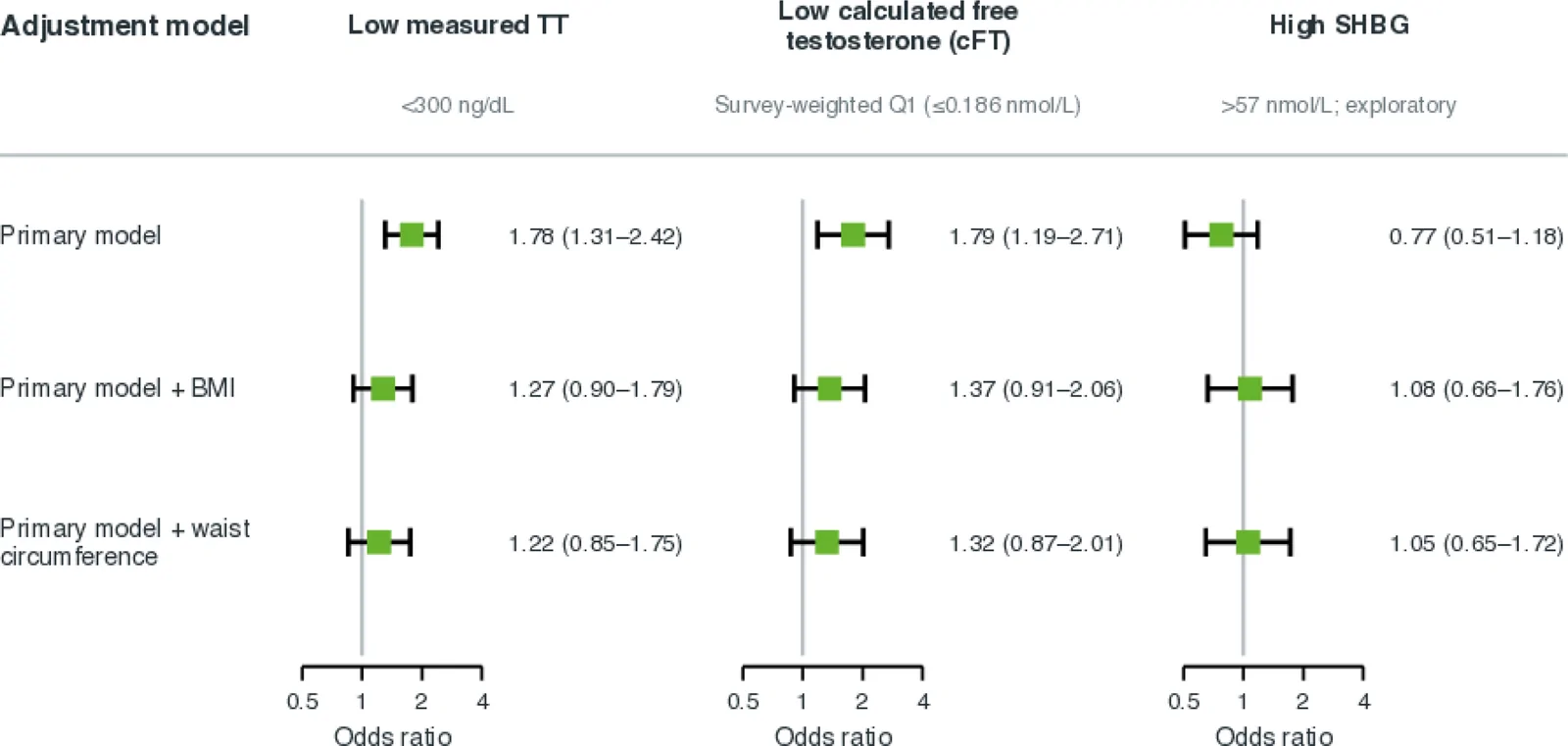
**Androgen Measures and Prevalent Advanced Cardiovascular-Kidney-Metabolic Syndrome** Survey-weighted prevalence ORs for NHANES-adapted advanced CKM stages 3 to 4 are shown for low measured TT (<300 ng/dL), low cFT defined by the survey-weighted first quartile (≤0.186 nmol/L), and high SHBG (>57 nmol/L; exploratory).^27^ The primary model adjusted for flexible age, race and ethnicity, education, poverty-income ratio, smoking, alcohol use, physical activity, sedentary time, and National Health and Nutrition Examination Survey cycle. Body mass index and waist circumference were added separately. Squares indicate ORs, horizontal lines indicate 95% CIs, and the vertical line marks 1. Model-specific sample sizes and numbers of advanced-CKM cases are shown. BMI = body mass index; other abbreviations as in Figure 1.

Associations of cFT with advanced CKM varied according to exposure definition. Continuous cFT per 0.1 nmol/L was not associated with advanced CKM (OR: 1.01; 95% CI: 0.79-1.30; *P* = 0.90). In contrast, low cFT defined by the survey-weighted first quartile (OR: 1.79; 95% CI: 1.19-2.71; *P* = 0.010) or by a threshold below 0.220 nmol/L (OR: 1.77; 95% CI: 1.24-2.54; *P* = 0.005) was associated with higher odds of advanced CKM. Neither the continuous TT-to-SHBG ratio nor its weighted first-quartile category was associated with advanced CKM in the primary model.

Low TT also showed nominal associations with stage 3, stage 4, very-high-risk CKD, and clinical CVD; however, none met the within-family FDR criterion (q < 0.05). Results for the PREVENT-defined component were imprecise (Supplemental Table 3).

### Androgen measures and mortality

During 21,878.1 person-years of follow-up, 316 all-cause deaths and 105 cardiovascular deaths occurred. The survey-weighted median follow-up was 4.67 years [Q1-Q3: 3.75-5.83]. Available-sample mortality models included up to 3,869 men and 266 all-cause deaths.

Low TT was not associated with all-cause mortality in the available-sample model (HR: 1.06; 95% CI: 0.86-1.31; *P* = 0.60). Low cFT defined by the weighted first quartile was associated with a higher but imprecisely estimated mortality hazard (HR: 1.45; 95% CI: 0.97-2.18; *P* = 0.072). Among the exploratory joint TT-SHBG phenotypes, the low-TT/high-SHBG group was associated with higher all-cause mortality compared with the normal-TT/nonhigh-SHBG group (HR: 2.06; 95% CI: 1.18-3.58; *P* = 0.011), whereas low TT alone and high SHBG alone were not associated with mortality (Figure 3). This association did not meet the FDR criterion (q < 0.05).

**Figure 3:**
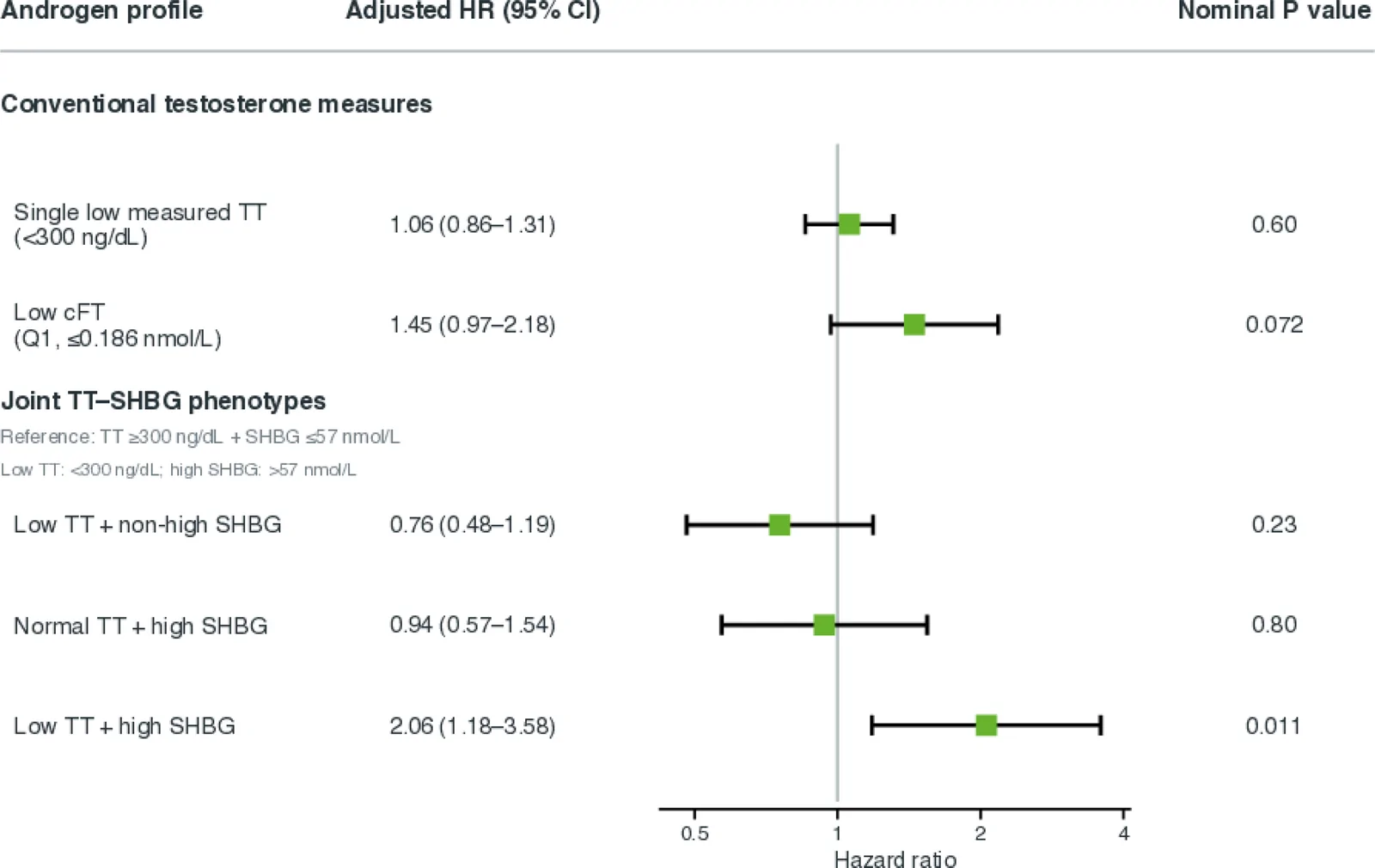
**Androgen Profiles and All-Cause Mortality** Survey-weighted Cox HRs for all-cause mortality are shown from available-sample models adjusted for flexible age, race and ethnicity, education, poverty-income ratio, smoking, alcohol use, physical activity, sedentary time, and National Health and Nutrition Examination Survey cycle, without cardiovascular-kidney-metabolic syndrome stage adjustment. Exposures include low measured TT (<300 ng/dL), low cFT (≤0.186 nmol/L), and mutually exclusive TT-SHBG phenotypes. Models included 3,869 men and 266 deaths, except the cFT model (3,865 men and 266 deaths). The low-TT/high-SHBG phenotype comprised 113 men and 42 deaths and showed a nominal association with mortality that did not survive Benjamini-Hochberg false discovery rate correction (q = 0.093). Joint-phenotype analyses were exploratory. cFT = calculated free testosterone; other abbreviations as in Figure 1.

**Central Illustration:**
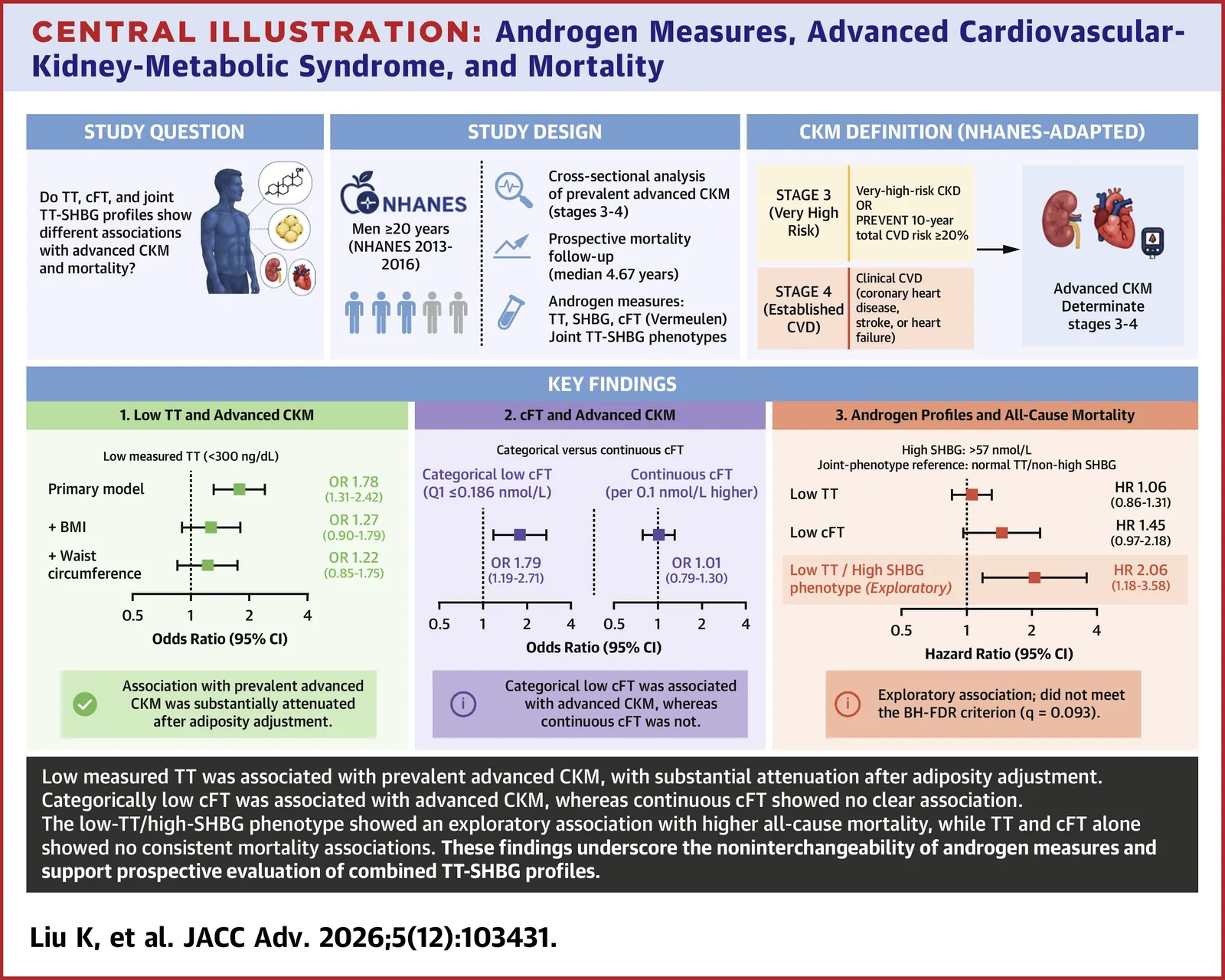
**Androgen Measures, Advanced Cardiovascular-Kidney-Metabolic Syndrome, and Mortality** Among U.S. men aged ≥20 years in NHANES 2013 to 2016, associations of androgen measures with prevalent advanced cardiovascular-kidney-metabolic (CKM) syndrome and subsequent all-cause mortality differed according to the measure and its analytic representation. Low measured total testosterone (TT) was associated with prevalent advanced CKM, but the association was substantially attenuated after adjustment for body mass index or waist circumference. Categorically low calculated free testosterone (cFT) was associated with advanced CKM, whereas continuous cFT was not. In exploratory mortality analyses, the joint low-TT/high–sex hormone-binding globulin (SHBG) phenotype was associated with higher all-cause mortality, although this finding did not meet the Benjamini-Hochberg false discovery rate criterion. These findings highlight the importance of adiposity context, analytic representation, and combined androgen profiles when interpreting androgen measures in CKM. BH-FDR = Benjamini-Hochberg false discovery rate; BMI = body mass index; CKD = chronic kidney disease; CVD = cardiovascular disease; NHANES = National Health and Nutrition Examination Survey.

Mortality estimates were attenuated after restriction to participants with determinate CKM stage. Most of the attenuation occurred with sample restriction, with comparatively smaller changes after additional adjustment for CKM stage. Model-specific sample sizes, deaths, and complete comparisons across the available-sample, CKM-complete, and CKM-adjusted estimands are presented in the Supplemental Material. Cardiovascular mortality estimates were imprecise, and none of the evaluated associations met nominal statistical significance (Supplemental Table 4).

### Flexible and sensitivity analyses

Flexible models suggested possible nonlinear associations for the TT-to-SHBG ratio and cFT, but these patterns were attenuated after restriction to the weighted 1st–99th percentile of each exposure distribution. Complete spline results, including overall and nonlinear tests, are provided in the Supplemental Material.

Sensitivity analyses incorporating measured albumin, chronic illness, functional limitation, relevant medications, examination session, and exclusion of early deaths generally attenuated the TT-to-SHBG ratio and low-TT/high-SHBG phenotype estimates. Adjustment for examination session and restriction to morning samples did not materially strengthen the association between low TT and mortality. Phenotype-specific event counts, cluster-influence analyses, complete-case exclusions, and comparisons between included and excluded participants are reported in Supplemental Tables 5 to 8 and Supplemental Figure 1.

## Discussion

In this nationally representative study of U.S. adult men, androgen measures showed distinct associations with prevalent CKM burden and mortality. A single low measured TT concentration was associated with greater odds of prevalent advanced NHANES-adapted CKM, although this association was substantially attenuated after adjustment for BMI or waist circumference. Neither low TT nor cFT showed a consistent association with all-cause mortality. In exploratory analyses, the low-TT/high-SHBG phenotype was associated with approximately 2-fold higher all-cause mortality, whereas low TT alone and high SHBG alone were not. Together, these findings suggest that conventional testosterone measures and combined TT-SHBG profiles may reflect complementary dimensions of adiposity-related cardiometabolic burden and broader systemic health (Central Illustration).

The attenuation of the low-TT–advanced-CKM association after adjustment for adiposity is central to interpreting the CKM findings, but it does not eliminate their potential clinical relevance. Adiposity is closely connected to both circulating testosterone and the CKM continuum. Excess adiposity may lower TT through insulin resistance, inflammatory signaling, altered hypothalamic-pituitary-gonadal function, and changes in testosterone metabolism, whereas overall and central adiposity are major components of the metabolic burden captured by CKM staging.^9^, ^11^, ^12^, ^16^, ^18^ In shared-complete-case analyses, adjustment for BMI or waist circumference produced similar attenuation without changing the participants included, supporting a substantive role of adiposity rather than an effect attributable to sample restriction.

These findings suggest that low measured TT may serve as an accessible signal of the adiposity-related metabolic burden accompanying advanced CKM, even though the association was not independent of BMI or waist circumference. A low TT concentration may therefore draw attention to the broader clinical context in which it occurs, including obesity, central adiposity, glycemic abnormalities, kidney dysfunction, and cardiovascular health, rather than being interpreted solely as an isolated endocrine finding. Because TT and CKM status were assessed at the same examination, the temporal direction of this relationship cannot be determined.

The cFT findings further illustrate the importance of measurement choice. Continuous cFT was not associated with advanced CKM, whereas selected categorical definitions produced positive associations, suggesting that the observed relationship varied according to how cFT was represented. In addition, cFT did not reproduce the mortality pattern observed for the low-TT/high-SHBG phenotype. These results support the view that TT, cFT, and combined TT-SHBG profiles characterize related but not interchangeable aspects of androgen physiology and systemic health.

The distinction between the TT-to-SHBG ratio and joint TT-SHBG phenotypes is particularly relevant. The ratio historically termed the free androgen index does not reliably estimate free testosterone in adult men, because it does not account for albumin binding or the nonlinear relationship between TT and SHBG.^20^, ^21^ Moreover, a low ratio may arise from low TT, high SHBG, or both, combining potentially different physiological states within the same value. Joint classification preserves the separate contributions of TT and SHBG and may therefore reveal heterogeneity that is obscured by a ratio or by cFT alone.

The low-TT/high-SHBG phenotype may represent a distinct systemic-vulnerability profile. Low TT can accompany adiposity, insulin resistance, aging, chronic disease, and reduced physical health, whereas SHBG reflects a different and sometimes opposing range of physiological influences.^9^, ^15^, ^22^, ^23^, ^24^, ^25^, ^26^ Lower SHBG is commonly associated with hepatic insulin resistance, adiposity, and metabolic dysfunction.^11^, ^12^, ^13^, ^14^, ^16^ In contrast, higher SHBG in older or chronically ill men may coexist with low TT, altered nutritional status, medication exposure, functional impairment, or broader systemic illness.^15^, ^17^, ^22^, ^25^, ^26^ The concurrence of low TT and high SHBG may therefore reflect the convergence of 2 distinct hormonal and health-related signals rather than simply indicating reduced free testosterone.

The concentration of the mortality association in the combined low-TT/high-SHBG group, rather than in the low-TT-only or high-SHBG-only groups, supports this interpretation. The approximately 2-fold estimate raises the possibility that combined TT-SHBG profiling captures systemic-health heterogeneity not apparent from conventional TT or cFT measures alone. No clear association was observed with cardiovascular mortality. This pattern may indicate that the all-cause mortality signal relates to broader systemic health rather than to cardiovascular mortality specifically, although the limited number of cardiovascular deaths reduced statistical precision.

The CKM framework provides a clinically relevant setting in which to examine these androgen patterns because it integrates adiposity, metabolic dysfunction, CKD, predicted cardiovascular risk, and established CVD.^1^, ^2^ Previous studies have generally evaluated testosterone and SHBG in relation to individual metabolic disorders, cardiovascular outcomes, or mortality.^11^, ^12^, ^13^, ^14^, ^16^, ^22^, ^23^, ^24^, ^25^, ^26^ By examining several androgen measures within CKM staging, the present study suggests 2 potentially complementary patterns: low TT may primarily accompany adiposity-related CKM burden, whereas the low-TT/high-SHBG phenotype may reflect broader systemic vulnerability relevant to mortality.

From a clinical perspective, these findings support contextual interpretation of androgen measurements. A single TT concentration below 300 ng/dL may accompany a greater burden of adiposity-related metabolic, kidney, and cardiovascular abnormalities and may provide a cue to assess overall CKM health. This interpretation does not imply that low TT independently predicts CKM or that routine testosterone testing should be used for CKM screening. A single low-TT value also should not be equated with clinical hypogonadism, which requires compatible symptoms and confirmation with repeat morning measurements.^28^, ^29^, ^30^

The low-TT/high-SHBG profile may identify a different dimension of systemic health that warrants further mechanistic and prognostic study. Its potential relevance lies in highlighting the joint hormonal context rather than replacing established CKM assessment or directing testosterone treatment. The present study did not evaluate discrimination, calibration, risk reclassification, or clinical decision utility and therefore cannot establish that combined TT-SHBG profiles improve risk prediction. Nevertheless, the observed phenotype provides a biologically and clinically plausible target for prospective investigation.

Strengths of this study include the use of a nationally representative sample, standardized examination and laboratory procedures, design-based survey analyses, linked mortality follow-up, explicit PREVENT eligibility criteria, and CKM staging that did not classify missing components as absent. The analysis incorporated albumin-based cFT, distinguished the TT-to-SHBG ratio from joint TT-SHBG phenotypes, and separated the effects of adiposity adjustment, CKM sample restriction, and CKM-stage adjustment. Shared-complete-case analyses further helped distinguish changes attributable to covariate adjustment from those caused by changes in the analytic population.

### Study limitations

Hormone concentrations were measured once, without symptom assessment or repeat morning confirmation, and the timing of collection was not uniform. Luteinizing hormone, follicle-stimulating hormone, prolactin, and sufficiently detailed clinical information were unavailable to determine the etiology of low TT. The CKM analysis was cross-sectional and therefore could not establish temporality or exclude reverse causation. NHANES did not include the imaging or complete biomarker data required to identify the full spectrum of subclinical CVD, potentially resulting in underascertainment or misclassification of stage 3 CKM; clinical CVD was also based on self-report. Complete-case selection may have introduced selection bias. Cancer, inflammatory disorders, liver and thyroid disease, nutritional status, functional impairment, and medications affecting androgen physiology could influence both hormone profiles and mortality. Although several available illness, albumin, functional, and medication variables were examined, residual confounding remains possible. cFT was equation-derived rather than directly measured, and assay or equation error may have affected classification. The low-TT/high-SHBG phenotype was uncommon, and its mortality association weakened after restriction to participants with determinate CKM stage and in several sensitivity analyses. The association also did not meet the FDR criterion (q < 0.05) and should therefore be considered exploratory. Finally, the limited number of cardiovascular deaths reduced precision for cardiovascular-specific mortality analyses.

## Conclusion

A single low measured TT concentration was associated with greater prevalent advanced NHANES-adapted CKM burden, with substantial attenuation after adjustment for overall or central adiposity. Low TT may therefore serve as a clinical signal of adiposity-related CKM burden rather than an adiposity-independent risk marker. Conventional TT and cFT measures did not show consistent mortality associations, whereas the exploratory low-TT/high-SHBG phenotype was associated with higher all-cause mortality and may reflect broader systemic vulnerability. Prospective studies incorporating repeated morning hormone measurements, symptom assessment, validated or directly measured free testosterone, comprehensive characterization of chronic illness and frailty, and repeated CKM staging are needed to clarify the clinical and prognostic relevance of combined TT-SHBG profiles.

Perspectives **COMPETENCY IN MEDICAL KNOWLEDGE:** A single low measured total testosterone concentration may accompany greater adiposity-related CKM burden and should be interpreted within the broader metabolic, kidney, and cardiovascular context. The exploratory low-TT/high-SHBG phenotype may capture a distinct systemic-health profile associated with mortality.

**TRANSLATIONAL OUTLOOK:** Future prospective studies incorporating repeated morning hormone measurements, longitudinal CKM assessment, and comprehensive characterization of chronic illness and frailty are needed to determine whether combined TT-SHBG profiles provide reproducible prognostic information beyond established clinical factors.

## Funding support and author disclosures

This work was supported by the National Natural Science Foundation of China (Grant No. 82170460) and the Shanghai Top Priority Research Center Construction Project (Grant No. 2022ZZ01010). The authors have reported that they have no relationships relevant to the contents of this paper to disclose.
