# Supplementary material for: Androgen Imbalance, Cardiovascular-Kidney-Metabolic Syndrome Stages, and Mortality in Men
Source: JACC Adv. 2026 Nov 5;5(12):103431. doi: 10.1016/j.jacadv.2026.103431 (PMC13761257; doi:10.1016/j.jacadv.2026.103431)
Supplement: Supplementary file 1 [file mmc1.docx]

**Supplementary Material**

**Androgen Imbalance, Cardiovascular-Kidney-Metabolic Syndrome Stages, and Mortality in Men**

# Contents

Supplementary Methods

S1. NHANES-adapted CKM staging and PREVENT implementation

S2. Androgen measures and collection conditions

S3. Stage- and component-specific CKM associations and morning-session sensitivity

S4. Cardiovascular mortality

S5. All-cause mortality models and sensitivity analyses

S6. Proportional-hazards diagnostics and time-specific estimates

S7. TT-SHBG phenotype outcomes, crude rates, influence, and interaction

S8. Missingness, sample flow, and selection comparison

S9. Simultaneous total-testosterone and SHBG models

S10. Correlations among continuous androgen measures

S11. Cross-classification of androgen categories by total testosterone

Supplementary Figure 1. Tail-restricted flexible mortality associations

# Supplementary Methods

## Complex Survey Design and Analytic Domains

All analyses incorporated the NHANES complex survey design, including sampling weights, strata, and primary sampling units (PSUs). The full two-cycle design was constructed with survey::svydesign(ids=~SDMVPSU, strata=~SDMVSTRA, weights=~combined weight, nest=TRUE) before restriction to adult men, hormone availability, outcome eligibility, or complete covariates. Analytic populations were then defined as survey domains so that the original strata and PSUs were retained. Lonely PSUs were handled using the survey package's adjust option.

The combined-cycle MEC weight was calculated as WTMEC2YR/2 for analyses based on the mobile examination center sample. Survey-weighted quasibinomial logistic models were fitted using survey::svyglm() for prevalent CKM outcomes. Mortality associations were estimated using fully design-based survey::svycoxph() models. All reported hazard ratios and 95% confidence intervals were derived from these design-based models rather than from ordinary Cox regression treating sampling weights as simple case weights.

## Proportional-Hazards Diagnostics

Because survey::svycoxph() does not directly provide the standard residual-based proportional-hazards test used for ordinary Cox models, covariate-identical survival::coxph() models were fitted as approximate diagnostic checks. Survey weights were normalized by their mean; the diagnostic models were stratified by SDMVSTRA and used robust variance clustered on the SDMVSTRA-by-SDMVPSU combination. Proportional hazards were evaluated with survival::cox.zph(transform="km"). These diagnostic fits were not used for effect estimation.

Under the post-review analysis rule, an unrounded exposure-specific proportional-hazards P value below 0.05 triggered descriptive design-based estimates for 0–3 years and for follow-up after 3 years, using the same covariates and analytic domain as the source model. Cardiovascular-mortality proportional-hazards diagnostics were not estimable with 75 model-complete deaths; the corresponding survey-weighted Cox estimates were retained unchanged.

## Influence and Missing-Data Assessment

For the uncommon low-TT/high-SHBG phenotype, single-cluster influence was assessed with a stratified JKn delete-one-PSU replicate design constructed before restriction to the exact model-complete domain. Each replicate deleted one PSU and compensated the remaining PSU or PSUs within its stratum. The replicate hazard-ratio range, maximum absolute change, directional consistency, and the phenotype participants and deaths in the deleted cluster were summarized as point-estimate influence diagnostics; these checks were not interpreted as proof of general stability or as resolving sparse-cell uncertainty.

Primary analyses used complete-case survey domains with model-specific sample sizes and cases or deaths. Multiple imputation was not performed. Missingness was summarized for major demographic, lifestyle, hormone, adiposity, albumin, CKM-stage, and mortality variables; sequential model exclusions and survey-weighted comparisons of included and excluded participants were also reported. Missing values were not treated as absence when CKM components were classified.

## Flexible Modeling and Tail Sensitivity

Flexible exposure models used restricted cubic splines with four knots at the survey-weighted 5th, 35th, 65th, and 95th percentiles and the survey-weighted median as the reference. The percentile scheme and reference rule were fixed in the post-review analysis plan and applied consistently across the corresponding flexible exposure-outcome analyses. Numerical knot locations were derived from the relevant weighted analysis domain rather than selected according to observed associations, statistical significance, or model fit. Overall and nonlinear tests used survey-design degrees of freedom.

Full-range flexible analyses retained the observed exposure values without winsorization. For TT-to-SHBG ratio and calculated free testosterone mortality models, additional sensitivity analyses restricted each exposure to its survey-weighted 1st–99th percentile range. Simultaneous TT-SHBG models used the same four-knot percentile scheme for both hormones.

## Relationships Among Androgen Measures

Exploratory descriptive analyses included 4,588 men aged ≥20 years with all four androgen measures available; four men with paired TT and SHBG measurements but missing albumin were excluded. Survey-weighted Spearman correlations used weighted mid-distribution ranks and the full survey design before domain restriction, with combined-cycle MEC weights (WTMEC2YR/2). Standard errors used a stratified delete-one-PSU jackknife with 60 replicates; ranks were recalculated in each replicate, and variance was centered on the full-sample estimate. Two-sided Wald t tests used 30 survey-design degrees of freedom. Correlation P values were nominal, without multiplicity adjustment, for these exploratory descriptive analyses. Cross-tabulations reported unweighted counts and survey-weighted row percentages. The androgen cutpoints used in the main analyses were retained, with unrounded values used for classification (Supplementary Tables S10 and S11).

# Supplementary Table S1. NHANES-Adapted CKM Staging and PREVENT Implementation

*NHANES operational criteria and PREVENT implementation used to derive CKM stages 0-4 and advanced CKM (stages 3-4).*

## Panel A. Hierarchical staging definition

| **Stage** | **Hierarchical criterion** | **NHANES operationalization** |
| --- | --- | --- |
| Stage 4 | Clinical cardiovascular disease | Self-reported coronary heart disease, angina, myocardial infarction, stroke, or heart failure. |
| Stage 3 | No stage 4 and very-high-risk CKD or PREVENT risk >=20% | Only observed kidney and PREVENT pathways were used; unavailable subclinical-CVD constructs were not imputed. |
| Stage 2 | No higher stage and at least one metabolic/kidney condition | Hypertension, diabetes, CKD, nonfasting triglycerides >=135 mg/dL, or operational metabolic syndrome. |
| Stage 1 | No higher stage and adverse adiposity/glycemia | Overweight/obesity, abdominal obesity, or prediabetes. |
| Stage 0 | All higher-stage and stage-1 components absent | Missing components were not recoded as absence. |
| Indeterminate | Observed data did not establish or exclude a stage | Tri-state component logic was used: present, absent, or missing. |

## Panel B. PREVENT applicability and stage-3 pathways

| **Item** | **Specification or result** |
| --- | --- |
| Observed stage-3/4 pathways | Clinical CVD and very-high-risk CKD were available; PREVENT was partly available. Imaging and a complete cardiac-biomarker panel were unavailable. |
| PREVENT eligibility | Total CVD (fatal/nonfatal ASCVD plus heart failure); Age 30-79 years, no known baseline ASCVD or heart failure, all required inputs observed and within published ranges |
| Published applicability ranges | Age 30–79 years; systolic blood pressure 90–200 mm Hg; total cholesterol 130–320 mg/dL; HDL cholesterol 20–100 mg/dL; eGFR 15–140 mL/min/1.73 m²; BMI 18.5 to <40 kg/m². |
| Out-of-range handling | No continuous input is truncated or clamped; any required input outside its published range yields missing PREVENT risk |
| Adults aged >=80 years | 272/4,592 (3.7% weighted); age was not capped in the primary staging definition. |
| Calculable PREVENT risk | 2,639/4,592 (60.4% weighted). |
| PREVENT risk >=20% | 209 participants among 2,639 with calculable PREVENT risk; 5.9% weighted in the full paired-hormone domain. |
| Any required input out of range | 675/4,449 (13.5% weighted); risk was set to missing, not truncated. |
| Stage-3 entry routes | PREVENT only: 183/290 (69.8% weighted); very-high-risk CKD only: 81/290 (24.3%); both: 26/290 (5.9%). |

Note. Advanced CKM was defined as stages 3-4 versus stages 0-2 among participants with determinate status. PREVENT risk was calculated only within its published age and input ranges; out-of-range inputs were treated as missing rather than truncated. CKD indicates chronic kidney disease; CVD, cardiovascular disease; PREVENT, Predicting Risk of Cardiovascular Disease EVENTS.

# Supplementary Table S2. Androgen Measures and Collection Conditions

*Definitions, analytic cutpoints, diagnostic limitations, and examination-session and fasting characteristics.*

## Panel A. Measures and diagnostic boundaries

| **Measure or construct** | **Availability** | **Definition and boundary** |
| --- | --- | --- |
| Single total testosterone measurement | Available; 5,000/5,505 | One NHANES serum total testosterone measurement, ng/dL. Use single low measured total testosterone terminology only |
| Sex hormone-binding globulin | Available; 4,592/5,505 | One NHANES serum SHBG measurement, nmol/L. Directly modeled continuously and jointly with total testosterone |
| TT-to-SHBG ratio | Derived; 4,592/5,505 | 100 × total testosterone (nmol/L) / SHBG (nmol/L). Exploratory ratio |
| Albumin-based calculated free testosterone | Derived; 4,588/5,505 | Vermeulen mass-action calculation using measured total testosterone, SHBG, and serum albumin. Equation-derived research measure |
| Compatible symptoms | Unavailable | No symptom inventory aligned with an endocrine diagnostic evaluation |
| Repeat morning testosterone | Unavailable | No repeat morning measurement for biochemical confirmation |
| LH, FSH, and prolactin | Unavailable | Etiology of a low testosterone concentration could not be classified as primary or secondary gonadal dysfunction. |

## Panel B. Cutpoints and collection conditions

| **Item** | **Definition or result** |
| --- | --- |
| Single low measured TT | <300 ng/dL; epidemiologic single-measure cutoff, not a diagnosis of hypogonadism. |
| Low TT-to-SHBG ratio | Weighted Q1: 26.41 (100 × TT [nmol/L] / SHBG [nmol/L]; derivation N=4,592); exploratory ratio, not free testosterone. |
| Low calculated free testosterone | Weighted Q1: 0.186 nmol/L (derivation N=4,588). |
| Examination session | Morning 2,192/4,592 (48.5%); afternoon 1,704/4,592 (35.4%); evening 696/4,592 (16.1%). |
| Fasting | >=8 hours: 2,244/4,592 (49.0%); morning plus >=8 hours: 2,035/4,592 (45.3%). |
| High-SHBG category | >57 nmol/L corresponds to the NHANES adult-male assay reference-range upper limit and retains the originally submitted exploratory cutoff; it is not an age-specific or outcome-validated clinical threshold. |

Note. cFT was calculated using the Vermeulen mass-action equation with measured TT, SHBG, and albumin and was independently checked against numerical mass-balance solutions. cFT indicates calculated free testosterone; SHBG, sex hormone-binding globulin; TT, total testosterone.

# Supplementary Table S3. Stage- and Component-Specific CKM Associations and Morning-Session Sensitivity

*Survey-weighted stage- and component-specific associations and the post-review morning-session sensitivity for the principal advanced-CKM analysis are shown.*

## Panel A. Stage- and component-specific associations

| **Exposure** | **N** | **Cases** | **OR (95% CI)** | **P value** | **BH-FDR q** |
| --- | --- | --- | --- | --- | --- |
| **Stage 3 versus stages 0-2; stage 4 excluded** | | | | | |
| Calculated free testosterone weighted Q1 | 2,930 | 250 | 1.28 (0.70-2.36) | 0.38 | 0.50 |
| SHBG per 10 nmol/L | 2,930 | 250 | 0.92 (0.84-1.01) | 0.079 | 0.28 |
| Single low measured total testosterone (<300 ng/dL) | 3,180 | 272 | 1.65 (1.00-2.73) | 0.049 | 0.23 |
| **Stage 4 versus stages 0-2; stage 3 excluded** | | | | | |
| Calculated free testosterone weighted Q1 | 3,148 | 468 | 2.04 (1.24-3.35) | 0.009 | 0.13 |
| SHBG per 10 nmol/L | 3,148 | 468 | 0.96 (0.88-1.04) | 0.25 | 0.44 |
| Single low measured total testosterone (<300 ng/dL) | 3,425 | 517 | 1.75 (1.19-2.57) | 0.009 | 0.13 |
| **Very-high-risk chronic kidney disease** | | | | | |
| Calculated free testosterone weighted Q1 | 3,833 | 146 | 1.43 (0.80-2.57) | 0.20 | 0.39 |
| SHBG per 10 nmol/L | 3,833 | 146 | 0.90 (0.84-0.98) | 0.017 | 0.14 |
| Single low measured total testosterone (<300 ng/dL) | 4,178 | 156 | 1.77 (1.05-2.98) | 0.035 | 0.18 |
| **PREVENT 10-year total-CVD risk >=20%** | | | | | |
| Calculated free testosterone weighted Q1 | 2,259 | 179 | 1.03 (0.51-2.05) | 0.93 | 0.93 |
| SHBG per 10 nmol/L | 2,259 | 179 | 0.88 (0.78-0.99) | 0.033 | 0.18 |
| Single low measured total testosterone (<300 ng/dL) | 2,452 | 198 | 1.66 (0.87-3.18) | 0.11 | 0.29 |
| **Clinical cardiovascular disease** | | | | | |
| Calculated free testosterone weighted Q1 | 3,856 | 468 | 1.68 (1.13-2.49) | 0.015 | 0.14 |
| SHBG per 10 nmol/L | 3,860 | 468 | 0.98 (0.91-1.05) | 0.50 | 0.61 |
| Single low measured total testosterone (<300 ng/dL) | 4,209 | 517 | 1.39 (1.05-1.83) | 0.026 | 0.18 |

## Panel B. Post-review morning-session sensitivity for the principal CKM analysis

| **Exposure** | **Specification** | **N/cases** | **OR (95% CI)** | **P value** |
| --- | --- | --- | --- | --- |
| Single low measured total testosterone (<300 ng/dL) | Prevalent advanced CKM; morning examination session | 1,626/354 | 2.87 (1.56–5.28) | 0.003 |

Note. This post-review collection-timing sensitivity used the morning-session model-complete CKM sample and reports N/cases and an OR; it is not part of a multiplicity family and no q value was assigned. OR indicates odds ratio.

Note. Models incorporated the NHANES survey design and adjusted for flexible age, race/ethnicity, education, poverty-income ratio, smoking, alcohol use, physical activity, sedentary time, and NHANES cycle. Stage 4 and clinical cardiovascular disease overlap by definition. Overall advanced-CKM estimates are presented in Figure 2. CKD indicates chronic kidney disease; CVD, cardiovascular disease; SHBG, sex hormone-binding globulin; TT, total testosterone.

# Supplementary Table S4. Cardiovascular Mortality

*Survey-weighted cardiovascular-mortality associations are shown for four selected androgen measures.*

| **Exposure** | **N** | **Deaths** | **HR (95% CI)** | **P value** | **BH-FDR q** |
| --- | --- | --- | --- | --- | --- |
| Single low measured total testosterone (<300 ng/dL) | 3,395 | 75 | 0.89 (0.52-1.52) | 0.68 | 0.82 |
| Low TT-to-SHBG ratio (weighted Q1) | 3,395 | 75 | 0.95 (0.67-1.35) | 0.78 | 0.82 |
| Calculated free testosterone weighted Q1 | 3,395 | 75 | 1.21 (0.79-1.85) | 0.39 | 0.82 |
| Low TT plus high SHBG phenotype | 3,395 | 75 | 0.90 (0.49-1.66) | 0.75 | 0.82 |

Note. Models incorporated the NHANES survey design and adjusted for flexible age, race/ethnicity, education, poverty-income ratio, smoking, alcohol use, physical activity, sedentary time, NHANES cycle, and CKM stage. The model-complete analysis contained 75 cardiovascular deaths; no association met nominal or BH-FDR significance. HR indicates hazard ratio.

# Supplementary Table S5. All-Cause Mortality Models and Sensitivity Analyses

*Available-sample, same-sample, and CKM-stage-conditioned estimates are shown with selected sensitivity analyses.*

## Panel A. Available-sample, CKM-complete same-sample, and CKM-stage-conditioned models

| **Estimand** | **N/deaths** | **HR (95% CI)** | **P value** |
| --- | --- | --- | --- |
| **Single low measured TT (<300 ng/dL)** | | | |
| Available-sample model | 3,869/266 | 1.06 (0.86–1.31) | 0.60 |
| CKM-complete same-sample model | 3,395/199 | 1.12 (0.85–1.46) | 0.42 |
| CKM-stage-conditioned model | 3,395/199 | 1.03 (0.78–1.37) | 0.83 |
| **Low TT-to-SHBG ratio (weighted Q1)** | | | |
| Available-sample model | 3,869/266 | 1.77 (1.17–2.69) | 0.007 |
| CKM-complete same-sample model | 3,395/199 | 1.46 (0.93–2.28) | 0.098 |
| CKM-stage-conditioned model | 3,395/199 | 1.35 (0.86–2.11) | 0.19 |
| **Calculated free testosterone (weighted Q1)** | | | |
| Available-sample model | 3,865/266 | 1.45 (0.97–2.18) | 0.072 |
| CKM-complete same-sample model | 3,395/199 | 1.20 (0.74–1.94) | 0.45 |
| CKM-stage-conditioned model | 3,395/199 | 1.05 (0.66–1.67) | 0.84 |
| **Low TT plus high SHBG phenotype** | | | |
| Available-sample model | 3,869/266 | 2.06 (1.18–3.58) | 0.011 |
| CKM-complete same-sample model | 3,395/199 | 1.74 (1.04–2.90) | 0.036 |
| CKM-stage-conditioned model | 3,395/199 | 1.58 (1.02–2.43) | 0.038 |

Note. Available-sample total associations used exposure-specific complete cases without CKM-stage adjustment. Same-sample total and CKM-stage-conditioned models used identical CKM-complete participants; only the latter added CKM stage. Within the all-cause mortality androgen-category/phenotype multiplicity family, the available-sample low TT-to-SHBG ratio and low-TT/high-SHBG phenotype associations each had a BH-FDR q value of 0.093, and the CKM-stage-conditioned low-TT/high-SHBG phenotype association had q=0.16. Same-sample estimates were interpretive comparisons and did not receive q values.

## Panel B. Selected sensitivity analyses

| **Exposure** | **Sensitivity specification** | **N/deaths** | **HR (95% CI)** | **P value** | **BH-FDR q** |
| --- | --- | --- | --- | --- | --- |
| **Morning examination-session restriction** | | | | | |
| Calculated free testosterone weighted Q1 | Morning examination session; MEC weight + CKM stage | 1,619/98 | 1.05 (0.56-1.95) | 0.88 | 0.99 |
| Low TT plus high SHBG phenotype | Morning examination session; MEC weight + CKM stage | 1,619/98 | 2.67 (1.14-6.21) | 0.023 | 0.58 |
| Low TT-to-SHBG ratio (weighted Q1) | Morning examination session; MEC weight + CKM stage | 1,619/98 | 1.34 (0.74-2.42) | 0.33 | 0.77 |
| Single low measured total testosterone (<300 ng/dL) | Morning examination session; MEC weight + CKM stage | 1,619/98 | 1.24 (0.74-2.09) | 0.41 | 0.88 |
| **First-year death exclusion** | | | | | |
| Calculated free testosterone weighted Q1 | Base model + CKM stage; deaths in first year excluded | 3,361/165 | 1.13 (0.66-1.92) | 0.66 | 0.99 |
| Low TT plus high SHBG phenotype | Base model + CKM stage; deaths in first year excluded | 3,361/165 | 1.86 (1.13-3.06) | 0.015 | 0.58 |
| Low TT-to-SHBG ratio (weighted Q1) | Base model + CKM stage; deaths in first year excluded | 3,361/165 | 1.36 (0.80-2.29) | 0.25 | 0.70 |
| Single low measured total testosterone (<300 ng/dL) | Base model + CKM stage; deaths in first year excluded | 3,361/165 | 1.08 (0.77-1.51) | 0.64 | 0.99 |
| **Illness burden, functional limitation, and albumin** | | | | | |
| Calculated free testosterone weighted Q1 | Base model + CKM stage, albumin, complete-case illness burden count, and self-reported functional-limitation proxy | 3,371/196 | 1.04 (0.65-1.65) | 0.88 | 0.99 |
| Low TT plus high SHBG phenotype | Base model + CKM stage, albumin, complete-case illness burden count, and self-reported functional-limitation proxy | 3,371/196 | 1.45 (0.88-2.41) | 0.15 | 0.70 |
| Low TT-to-SHBG ratio (weighted Q1) | Base model + CKM stage, albumin, complete-case illness burden count, and self-reported functional-limitation proxy | 3,371/196 | 1.18 (0.77-1.81) | 0.45 | 0.93 |
| Single low measured total testosterone (<300 ng/dL) | Base model + CKM stage, albumin, complete-case illness burden count, and self-reported functional-limitation proxy | 3,371/196 | 0.99 (0.72-1.36) | 0.95 | 0.99 |
| **Combined hormone-relevant medication exclusion** | | | | | |
| Calculated free testosterone weighted Q1 | Base model + CKM stage; exclude all identified hormone-relevant medication users | 3,118/145 | 0.93 (0.52-1.64) | 0.79 | 0.99 |
| Low TT plus high SHBG phenotype | Base model + CKM stage; exclude all identified hormone-relevant medication users | 3,118/145 | 1.49 (0.88-2.52) | 0.13 | 0.70 |
| Low TT-to-SHBG ratio (weighted Q1) | Base model + CKM stage; exclude all identified hormone-relevant medication users | 3,118/145 | 1.12 (0.69-1.84) | 0.64 | 0.99 |
| Single low measured total testosterone (<300 ng/dL) | Base model + CKM stage; exclude all identified hormone-relevant medication users | 3,118/145 | 1.04 (0.63-1.70) | 0.89 | 0.99 |

Note. Base covariates were flexible age, race/ethnicity, education, poverty-income ratio, smoking, alcohol use, physical activity, sedentary time, and NHANES cycle; all displayed sensitivity models also included CKM stage. The illness specification included serum albumin, cancer, chronic liver disease, thyroid disease, chronic lung disease, an arthritis proxy, and a functional-limitation proxy derived from PFQ049, PFQ051, PFQ054, PFQ057, PFQ059, and PFQ090. The combined medication exclusion removed current users of opioids, systemic glucocorticoids, testosterone/anabolic agents, antiandrogen or androgen-deprivation therapy, 5-alpha-reductase inhibitors, and spironolactone. BH-FDR q values were calculated within the exploratory mortality-sensitivity family. The additional panels below report their own model specifications and denominators. cFT indicates calculated free testosterone; CKM, cardiovascular-kidney-metabolic syndrome.

## Panel C. Adiposity-adjusted TT-to-SHBG ratio mortality estimates

| **Exposure** | **Specification** | **N/deaths** | **HR (95% CI)** | **P value** | **BH-FDR q** |
| --- | --- | --- | --- | --- | --- |
| Low TT-to-SHBG ratio (weighted Q1) | CKM stage; shared complete cases | 3,317/172 | 1.29 (0.81–2.08) | 0.28 | 0.70 |
| Low TT-to-SHBG ratio (weighted Q1) | CKM stage + BMI; shared complete cases | 3,317/172 | 1.29 (0.81–2.07) | 0.29 | 0.70 |
| Low TT-to-SHBG ratio (weighted Q1) | CKM stage + waist circumference; shared complete cases | 3,317/172 | 1.29 (0.81–2.07) | 0.29 | 0.70 |

Note. All three ratio models used the same shared adiposity-complete sample and included CKM stage; BMI and waist circumference were added separately. HR indicates hazard ratio.

## Panel D. Fixed-albumin cFT mortality sensitivity

| **Exposure** | **Specification** | **N/deaths** | **HR (95% CI)** | **P value** | **BH-FDR q** |
| --- | --- | --- | --- | --- | --- |
| Fixed-albumin cFT weighted Q1 | Available-sample model; no CKM stage | 3,865/266 | 1.50 (1.07–2.12) | 0.019 | 0.58 |
| Fixed-albumin cFT weighted Q1 | CKM-stage-conditioned model | 3,395/199 | 1.10 (0.71–1.70) | 0.68 | 0.99 |

Note. cFT was recalculated with albumin fixed at 43 g/L. The available-sample model did not adjust for CKM stage; the conditioned model did. cFT indicates calculated free testosterone; HR, hazard ratio.

# Supplementary Table S6. Proportional-Hazards Diagnostics and Time-Specific Estimates

*Exposure-specific proportional-hazards tests and the corresponding time-specific estimates are shown for selected all-cause mortality measures.*

## Panel A. All-cause mortality proportional-hazards diagnostics

| **Exposure** | **Available total** | **Same-sample total** | **CKM-stage conditioned** |
| --- | --- | --- | --- |
| Single low measured total testosterone (<300 ng/dL) | 3,869/266; 0.25 (0.11) | 3,395/199; 0.31 (0.082) | 3,395/199; 0.33 (0.086) |
| Calculated free testosterone weighted Q1 | 3,865/266; 0.090 (0.088) | 3,395/199; 0.040 (0.073) | 3,395/199; 0.050 (0.082) |
| Low TT-to-SHBG ratio (weighted Q1) | 3,869/266; 0.028 (0.089) | 3,395/199; 0.037 (0.087) | 3,395/199; 0.039 (0.098) |
| Low TT plus high SHBG phenotype | 3,869/266; 0.17 (0.12) | 3,395/199; 0.34 (0.088) | 3,395/199; 0.41 (0.099) |

Note. Each cell shows N/deaths; exposure-specific PH P value (global model PH P value). Proportional-hazards tests were obtained from normalized-weight survival::coxph() fits and should be interpreted as approximate diagnostics; they were not used to estimate the reported HRs and 95% CIs, which came from design-based survey::svycoxph() models. Cardiovascular-mortality PH diagnostics were not estimable with 75 model-complete deaths; the survey-weighted Cox estimates in Table S4 were unchanged.

## Panel B. Time-specific estimates for PH signals

| **Exposure** | **Interval** | **Available total** | **Same-sample total** | **CKM-stage conditioned** |
| --- | --- | --- | --- | --- |
| Low TT-to-SHBG ratio (weighted Q1) | 0–3 years | 3,869/163; 1.55 (0.85-2.83); P=0.15 | 3,395/119; 1.24 (0.60-2.57); P=0.57 | 3,395/119; 1.15 (0.54-2.43); P=0.72 |
| Low TT-to-SHBG ratio (weighted Q1) | >3 years | 3,670/103; 2.16 (0.94-4.97); P=0.070 | 3,244/80; 1.86 (0.75-4.57); P=0.18 | 3,244/80; 1.73 (0.75-3.97); P=0.20 |
| Calculated free testosterone weighted Q1 | 0–3 years | — | 3,395/119; 1.08 (0.66-1.77); P=0.75 | 3,395/119; 0.93 (0.55-1.57); P=0.79 |
| Calculated free testosterone weighted Q1 | >3 years | — | 3,244/80; 1.37 (0.64-2.92); P=0.42 | 3,244/80; 1.25 (0.64-2.46); P=0.51 |

Note. Each time-specific cell shows N/deaths; HR (95% CI); P value. An em dash indicates that the exposure-specific PH test did not trigger a time-specific estimate for that estimand. Displayed PH P values use two decimals when ≥0.10 and three when <0.10; trigger decisions used unrounded values. Under the post-review analysis rule, estimates were reported for 0-3 years and for follow-up after 3 years when the unrounded exposure-specific PH P value was <0.05; the same covariates and analytic domain as the source model were used.

# Supplementary Table S7. TT-SHBG Phenotype Outcomes, Crude Rates, Influence Diagnostics, and Formal Interaction

*Phenotype distribution and outcomes (Panel A), crude survey-weighted mortality rates (Panel B), sparse-phenotype influence diagnostics (Panel C), and the formal exploratory interaction (Panel D) are shown.*

## Panel A. Phenotype distribution and outcomes

| **Phenotype** | **N (weighted %)** | **All-cause deaths (weighted %)** | **CVD deaths (weighted %)** | **Person-years** | **Follow-up median [Q1, Q3], y** | **CKM-conditioned N/deaths** |
| --- | --- | --- | --- | --- | --- | --- |
| Overall | 4,582 (100.0%) | 316 (4.8%) | 105 (1.6%) | 21,878.1 | 4.667 [3.75, 5.833] | 3,395/199 |
| TT >=300 ng/dL and SHBG <=57 nmol/L | 2,371 (54.3%) | 81 (2.8%) | 32 (1.1%) | 11,626.7 | 4.833 [3.833, 5.917] | 1,842/55 |
| TT <300 ng/dL and SHBG <=57 nmol/L | 1,115 (24.4%) | 74 (4.2%) | 22 (1.2%) | 5,284.9 | 4.667 [3.667, 5.75] | 786/50 |
| TT >=300 ng/dL and SHBG >57 nmol/L | 983 (19.8%) | 119 (8.7%) | 36 (2.8%) | 4,535.7 | 4.5 [3.667, 5.667] | 702/71 |
| TT <300 ng/dL and SHBG >57 nmol/L | 113 (1.5%) | 42 (37.6%) | 15 (12.0%) | 430.8 | 4.25 [3.167, 5.167] | 65/23 |

Note. Counts, deaths, and observed person-years are unweighted; population and event percentages and follow-up summaries are survey weighted. High SHBG >57 nmol/L corresponds to the NHANES adult-male assay reference-range upper limit and retains the originally submitted exploratory cutoff; it is not an age-specific or outcome-validated clinical threshold. Continuous and flexible SHBG models provided the primary basis for interpretation.

## Panel B. Crude survey-weighted mortality rates

| **Phenotype** | **Crude survey-weighted all-cause rate per 1,000 person-years (95% CI)** | **Crude survey-weighted CVD rate per 1,000 person-years (95% CI)** |
| --- | --- | --- |
| Overall | 10.13 (8.52–11.73) | 3.44 (2.52–4.35) |
| TT ≥300 ng/dL and SHBG ≤57 nmol/L | 5.84 (4.11–7.56) | 2.31 (1.29–3.33) |
| TT <300 ng/dL and SHBG ≤57 nmol/L | 8.70 (6.28–11.12) | 2.55 (1.30–3.81) |
| TT ≥300 ng/dL and SHBG >57 nmol/L | 18.78 (13.19–24.37) | 6.07 (2.73–9.40) |
| TT <300 ng/dL and SHBG >57 nmol/L | 92.50 (62.58–122.41) | 29.48 (14.63–44.34) |

Note. Crude survey-weighted mortality rates are descriptive design-based event-to-person-time ratios with 95% CIs from survey::svyratio; adjusted mortality inference is based on the survey Cox models. CVD indicates cardiovascular disease.

## Panel C. Sparse-phenotype influence summary

| **Diagnostic** | **Result** |
| --- | --- |
| CKM-stage-conditioned model | N/deaths=3,395/199; HR=1.58 (1.02-2.43); P=0.038; q=0.16 |
| Delete-one-PSU replicate HR range | 1.46-1.75 |
| Maximum absolute HR change | 11.1% |
| Largest single PSU | 5 phenotype participants (7.7%) and 2 phenotype deaths (8.7%). |

Note. The influence diagnostic assesses whether one primary sampling unit dominates the point estimate; it does not remove sparse-cell uncertainty, residual confounding, multiplicity, or reverse etiology. The displayed q=0.16 is the BH-FDR value from the all-cause mortality androgen-category/phenotype family, not a separate correction of the influence diagnostic.

## Panel D. Formal low-TT by high-SHBG interaction

| **Outcome** | **Interaction term** | **N** | **HR (95% CI)** | **P value** |
| --- | --- | --- | --- | --- |
| All-cause mortality | Low measured TT by high-SHBG interaction | 3,395 | 2.72 (1.16-6.39) | 0.021 |

Note. The interaction was exploratory. Because the interaction family contained one test, its BH-FDR q value equaled its P value and is not shown as separate multiplicity protection. CVD indicates cardiovascular disease; HR, hazard ratio; PSU, primary sampling unit; SHBG, sex hormone-binding globulin; TT, total testosterone.

# Supplementary Table S8. Missingness, Sample Flow, and Selection Comparison

*Mortality sample flow, missingness in the paired hormone domain, and weighted comparisons of included and excluded participants are shown.*

## Panel A. Mortality sample flow

| **Step** | **Interpretation** | **N/deaths (%)** |
| --- | --- | --- |
| Raw mortality domain | Starting domain | 4,582/316 (100.0%) |
| Excluded by complete-case requirements for the available-sample model | Before fitting the available-sample no-CKM model | 713/50 (15.6%) |
| Available-sample no-CKM model | No CKM stage required | 3,869/266 (84.4%) |
| Newly excluded when CKM stage is required | CKM stage indeterminate | 474/67 (12.3%) |
| CKM-complete mortality model included | Used by both same-sample and CKM-stage-conditioned models | 3,395/199 (74.1%) |

## Panel B. Missingness for major analysis variables

| **Variable** | **Missing n/N (weighted %) in paired TT-SHBG domain** |
| --- | --- |
| Age | 0/4,592 (0.0% weighted) |
| Race and ethnicity | 0/4,592 (0.0% weighted) |
| Education | 2/4,592 (0.0% weighted) |
| Poverty-income ratio | 402/4,592 (7.4% weighted) |
| Smoking status | 5/4,592 (0.0% weighted) |
| Any alcohol use | 322/4,592 (6.2% weighted) |
| Moderate-to-vigorous physical activity | 27/4,592 (0.6% weighted) |
| Sedentary time | 30/4,592 (0.6% weighted) |
| Total testosterone | 0/4,592 (0.0% weighted) |
| Sex hormone-binding globulin | 0/4,592 (0.0% weighted) |
| Serum albumin | 4/4,592 (0.1% weighted) |
| Calculated free testosterone | 4/4,592 (0.1% weighted) |
| Body mass index | 57/4,592 (1.2% weighted) |
| Waist circumference | 213/4,592 (3.9% weighted) |
| Strict NHANES-adapted CKM stage | 618/4,592 (11.3% weighted) |
| Mortality follow-up | 8/4,592 (0.2% weighted) |

## Panel C. Included-versus-excluded comparison

| **Characteristic** | **Advanced-CKM model included** | **Advanced-CKM model excluded** | **Mortality model included** | **Mortality model excluded** |
| --- | --- | --- | --- | --- |
| Participants | 3,423 (87.4%) | 582 (12.6%) | 3,395 (77.6%) | 1,187 (22.4%) |
| Age | 46.2 (0.4) | 46.8 (1.0) | 46.2 (0.4) | 50.5 (0.6) |
| Total testosterone | 395.0 [302.0, 513.0] | 402.0 [295.0, 528.0] | 395.0 [302.0, 513.0] | 373.0 [274.0, 517.0] |
| Sex hormone-binding globulin | 37.2 [27.0, 52.5] | 37.5 [26.4, 53.5] | 37.2 [27.1, 52.5] | 39.7 [26.7, 57.3] |
| Body mass index | 28.6 (0.2) | 28.5 (0.3) | 28.6 (0.2) | 30.3 (0.4) |
| Waist circumference | 101.3 (0.5) | 100.1 (0.8) | 101.3 (0.5) | 104.9 (1.1) |
| Mortality follow-up | 4.83 [3.83, 5.92] | 4.42 [3.58, 5.67] | 4.83 [3.83, 5.92] | 4.50 [3.58, 5.67] |
| Advanced CKM | 720 (15.7%) | 129 (17.7%) | 717 (15.7%) | 129 (17.5%) |
| All-cause mortality | 202 (4.2%) | 38 (4.6%) | 199 (4.1%) | 117 (7.3%) |

Note. Missingness was summarized in the paired TT-SHBG domain (N=4,592). The mortality domain further excluded 10 participants: 8 with unavailable linked mortality/follow-up information and 2 with zero months of follow-up, yielding N=4,582. Panel A percentages used the 4,582-person mortality domain as the denominator, except for the 474 newly excluded participants, whose percentage used the 3,869-person available-sample model. The advanced-CKM comparison used 4,005 participants with determinate binary advanced-CKM status (3,423 model complete and 582 excluded); exact CKM stage was determinate for 3,974 participants. The mortality comparison used the 4,582-person mortality domain (3,395 model complete and 1,187 excluded). After fitting the available-sample no-CKM model, requiring CKM-stage determinacy additionally excluded 474 participants and 67 deaths. Continuous values are survey-weighted mean (SE) or median [Q1, Q3]; categorical values are unweighted n (survey-weighted %). Observed comparisons cannot exclude complete-case selection bias.

# Supplementary Table S9. Simultaneous Total-Testosterone and SHBG Models

*Total testosterone and SHBG were entered together as linear terms and as flexible spline terms.*

## Panel A. Simultaneous linear models

| **Outcome** | **Exposure term** | **Model** | **N/events** | **OR or HR (95% CI)** | **P value** | **BH-FDR q** |
| --- | --- | --- | --- | --- | --- | --- |
| Advanced CKM | Total testosterone per 100 ng/dL conditional on SHBG | Base model; TT and SHBG entered simultaneously | 3,423/720 | 0.92 (0.76-1.11) | 0.35 | 0.48 |
| Advanced CKM | SHBG per 10 nmol/L conditional on total testosterone | Base model; TT and SHBG entered simultaneously | 3,423/720 | 0.97 (0.87-1.09) | 0.62 | 0.72 |
| All-cause mortality | Total testosterone per 100 ng/dL conditional on SHBG | Base model; TT and SHBG simultaneous; no CKM | 3,869/266 | 0.90 (0.78-1.05) | 0.18 | 0.43 |
| All-cause mortality | SHBG per 10 nmol/L conditional on total testosterone | Base model; TT and SHBG simultaneous; no CKM | 3,869/266 | 1.10 (1.02-1.20) | 0.014 | 0.093 |
| All-cause mortality | Total testosterone per 100 ng/dL conditional on SHBG | Base model; TT and SHBG simultaneous + CKM stage | 3,395/199 | 0.94 (0.76-1.17) | 0.59 | 0.67 |
| All-cause mortality | SHBG per 10 nmol/L conditional on total testosterone | Base model; TT and SHBG simultaneous + CKM stage | 3,395/199 | 1.06 (0.94-1.20) | 0.31 | 0.46 |
| Cardiovascular mortality | Total testosterone per 100 ng/dL conditional on SHBG | Base model; TT and SHBG simultaneous + CKM stage | 3,395/75 | 0.88 (0.75-1.03) | 0.10 | 0.42 |
| Cardiovascular mortality | SHBG per 10 nmol/L conditional on total testosterone | Base model; TT and SHBG simultaneous + CKM stage | 3,395/75 | 1.01 (0.91-1.12) | 0.82 | 0.82 |

## Panel B. Simultaneous flexible models

| **Outcome** | **Spline term** | **Model** | **N/events** | **Overall P** | **Nonlinear P** | **BH-FDR q** |
| --- | --- | --- | --- | --- | --- | --- |
| Advanced CKM | Total testosterone | Base model; TT and SHBG four-knot splines simultaneous | 3,423/720 | 0.31 | 0.23 | 0.38 |
| Advanced CKM | SHBG | Base model; TT and SHBG four-knot splines simultaneous | 3,423/720 | 0.69 | 0.51 | 0.69 |
| All-cause mortality | Total testosterone | Base model no CKM; TT and SHBG splines simultaneous | 3,869/266 | 0.25 | 0.29 | 0.33 |
| All-cause mortality | SHBG | Base model no CKM; TT and SHBG splines simultaneous | 3,869/266 | 0.075 | 0.65 | 0.15 |
| All-cause mortality | Total testosterone | Base model + CKM stage; TT and SHBG splines simultaneous | 3,395/199 | 0.58 | 0.53 | 0.61 |
| All-cause mortality | SHBG | Base model + CKM stage; TT and SHBG splines simultaneous | 3,395/199 | 0.34 | 0.38 | 0.38 |

Note. Advanced CKM denotes stages 3–4. Models included both total testosterone and SHBG together with flexible age, race/ethnicity, education, poverty-income ratio, smoking, alcohol use, physical activity, sedentary time, and NHANES cycle; CKM-conditioned rows additionally included CKM stage. The coefficients therefore represent mutually adjusted associations and should not be interpreted as independent biological effects. Four-knot specifications used weighted 5th, 35th, 65th, and 95th percentiles. TT knots were 182, 333, 460.81, and 760 ng/dL (reference 392.35); SHBG knots were 15.78, 31.51, 46.33, and 89.42 nmol/L (reference 37.73).

# **Supplementary Table S10. Correlations Among Continuous Androgen Measures**

| **Measure** | **TT** | **cFT** | **SHBG** | **TT-to-SHBG ratio** |
| --- | --- | --- | --- | --- |
| TT | 1.000 | 0.723 (P <0.001) | 0.516 (P <0.001) | 0.277 (P <0.001) |
| cFT | 0.723 (P <0.001) | 1.000 | −0.145 (P <0.001) | 0.830 (P <0.001) |
| SHBG | 0.516 (P <0.001) | −0.145 (P <0.001) | 1.000 | −0.628 (P <0.001) |
| TT-to-SHBG ratio | 0.277 (P <0.001) | 0.830 (P <0.001) | −0.628 (P <0.001) | 1.000 |

Note. Values are survey-weighted Spearman ρ coefficients with P values in parentheses, based on 4,588 men aged ≥20 years in NHANES 2013–2016. Analyses accounted for the complex survey design and combined-cycle MEC weights; P values are nominal and two-sided. Calculation details are provided in Supplementary Methods. cFT and the TT-to-SHBG ratio share TT and SHBG as calculation components.

# **Supplementary Table S11. Cross-Classification of Androgen Categories by Total Testosterone**

Values are unweighted n (survey-weighted row %).

| **Other androgen category** | **TT (ng/dL)** | **N** | **Category absent n (%)** | **Category present n (%)** |
| --- | --- | --- | --- | --- |
| Low cFT | <300 | 1,230 | 389 (36.1) | 841 (63.9) |
| Low cFT | ≥300 | 3,358 | 2,927 (88.5) | 431 (11.5) |
| High SHBG | <300 | 1,230 | 1,117 (94.1) | 113 (5.9) |
| High SHBG | ≥300 | 3,358 | 2,374 (73.3) | 984 (26.7) |
| Low TT-to-SHBG ratio | <300 | 1,230 | 647 (59.6) | 583 (40.4) |
| Low TT-to-SHBG ratio | ≥300 | 3,358 | 2,603 (80.4) | 755 (19.6) |

Note. N is the unweighted row denominator. Category absent and category present refer to the first-column category, with percentages summing to 100% within each row. The same 4,588 men appear in each cross-tabulation. Low TT was defined as <300 ng/dL and high SHBG as >57 nmol/L. Low cFT and low TT-to-SHBG ratio were defined using the survey-weighted first-quartile cutpoints used in the main analyses (≤0.186 nmol/L and ≤26.41, respectively); unrounded cutpoints were used for classification. The ratio equals 100 × TT (nmol/L)/SHBG (nmol/L).

Abbreviations: cFT, calculated free testosterone; MEC, mobile examination center; NHANES, National Health and Nutrition Examination Survey; SHBG, sex hormone-binding globulin; TT, total testosterone.

# Supplementary Figure 1. Tail-Restricted Flexible Mortality Associations

Note. Sensitivity analyses refitted flexible survey-weighted all-cause mortality models adjusted for the demographic/lifestyle covariates and CKM stage after restricting each exposure to its weighted 1st–99th percentile range. Panel A shows the TT-to-SHBG ratio and panel B shows calculated free testosterone. Orange curves and bands show HRs and 95% CIs; vertical dotted lines indicate weighted-median reference values. Overall and nonlinear P values use survey-design degrees of freedom. The attenuated overall tests indicate sensitivity to observations in the distributional tails and are not interpreted as robust confirmation. cFT indicates calculated free testosterone; CKM, cardiovascular-kidney-metabolic syndrome; HR, hazard ratio; SHBG, sex hormone-binding globulin; TT, total testosterone.
